# Jaborandi in Whooping-Cough

**Published:** 1879-06-20

**Authors:** E. A. De Cailhol


### *ON THE USE OF JABORANDI IN WHOOPING COUGH.*

BY E. A. DE CAILHOL, M.D.

On March 28th, in the evening, I was called to see a young French boy, aged five years, stout, and of strong constitution, who had an attack of whooping cough.

His parents told me that for the last three or four days they had remarked that every evening between five and six o'clock the paroxysms seemed to increase in severity—that they grew worse and worse until they had become very alarming.

When I saw the child he was laboring under a violent paroxysm; his face was congested and cyanotic; the spasmodic cough threatening suffocation; skin dry; pulse 140 per minute, and temperature 104° F.

Knowing by long experience that the treatment of this affection is usually very troublesome, from the length of time it requires to effect a cure, I concluded to try an entirely new treatment with this well marked case.

Into two ounces of water I put sixty drops of the fluid extract of jaborandi (prepared by Parke, Davis & Co.). I intentionally mention the manufacturers, because in several instances I have remarked that their preparations were more powerful in their effects than those of some other firms. The medicine was directed to be given by the teaspoonful every ten or fifteen minutes until one-half of the mixture should be taken.

An hour or so later I visited the patient again and found him perspiring freely, he had already vomited large quantities of mucosities and was still vomiting; temperature and pulse quite normal. I remained for nearly an hour with the family on account of the vomiting being so *persistent*, and during that time he did not cough once. When I left he was sleeping soundly.

At half-past six o'clock next evening, I called again. The mother said that during the entire day the child had had no cough, but that between five and six o'clock the trouble had again commenced, but *not so soon* as the day before. The pulse was then 130, temperature 102° F., skin dry, face congested, etc.

I advised the administration of the remains of the mixture left from the preceding day, and to stop giving it as soon as the child should vomit, which directions were carried out.

When I called, two hours later, there was only a teaspoonful of the medicine left in the bottle. Pulse and temperature normal, and the child drenched in perspiration. He had again vomited much phlegm, and this time was profusely salivated.

Next day at about the same hour, the child coughed once or twice, but had no fever or other unpleasant symptom; the fourth day there was no cough at all, and since then he has been entirely well.

Have we a specific for whooping cough in jaborandi?—*Saint Louis Clin. Record.*

